# Association of Race With Post-operative Complications After Spinal Fusion in Children With Cerebral Palsy

**DOI:** 10.7759/cureus.32920

**Published:** 2022-12-25

**Authors:** Lauryn Brown, Denver Kraft, Aribah Shah, Christian Falgons, Theodore Quan, Alisa Malyavko, Sean Tabaie

**Affiliations:** 1 Orthopedic Surgery, George Washington University School of Medicine and Health Sciences, Washington DC, USA; 2 Orthopedic Surgery, Georgetown University School of Medicine, Washington DC, USA; 3 Orthopedic Surgery, Children's National Hospital, Washington DC, USA

**Keywords:** fusion, arthrodesis, race, complications, scoliosis, cerebral palsy (cp)

## Abstract

Introduction: Neuromuscular scoliosis in children with cerebral palsy (CP) can lead to debilitating difficulties with pain, ambulation, sitting, and respiratory or cardiac compromise. Spinal fusion can halt deformity progression, though the decision to undergo surgery involves an individualized risk-benefit assessment. The purpose of this study was to evaluate whether race is a risk factor for patients with CP to experience post-operative complications after spinal fusion.

Methods: This is a retrospective cohort analysis of a national database. Analyses methods include univariate analyses, multivariate regression models, and other ad-hoc tests.

Results: There were 3,081 pediatric patients with CP who underwent spinal fusion. Black patients had an increased risk of experiencing any post-operative complication compared to Caucasians (OR 1.322, 95% CI 1.099-1.590). Both Caucasian(p=0.005) and Black (p<0.001) races were risk factors for experiencing medical complications; Black patients had an increased risk compared to Caucasians (OR 1.373, 95% CI 1.130-1.667). Other races had a greater length of ICU stay than Caucasians (median {Mdn}=3.00 days vs Mdn=2.00, p=0.029), and longer total hospital stays than Caucasian and Black patients (Mdn=9.00 days vs Mdn=6.00 days vs Mdn=6.00 days, p<0.001).

Conclusion: Race is an independent risk factor for pediatric patients with CP to experience medical complications following spinal fusion surgery, with Black patients having an increased risk compared to Caucasians. Further, other races were found to have significantly longer ICU and total hospital length of stay. This study is the first to present race as a risk factor for children with CP to experience increased post-operative complications following spinal fusion and will be valuable in understanding their individualized peri-operative courses and risks.

## Introduction

Cerebral palsy (CP) encompasses a heterogeneous group of neuromuscular conditions caused by perinatal injuries to the developing brain. Depending on severity, children with CP exhibit varying degrees of abnormal muscle tone, control, and function. Among the motor and postural consequences, scoliosis is present in 21-64% of patients with CP [1,2]. The likelihood of developing scoliosis increases with severity of CP, as measured by the Gross Motor Function Classification System (GMFCS) scale [3]. This scale ranges from I (ambulating without limitations with advanced motor skill deficiencies) to V (mobility severely limited even with assistive devices). Children with a GMFCS level of IV-V have an approximate 50% risk of developing scoliosis by 18 years of age [4]. There exists an even higher risk of scoliosis in patients with CP with total body involvement combined with marked mental disability [2,5].

Without intervention, neuromuscular scoliosis can continue to progress into adulthood, further limiting ambulation, balance, and postured sitting [6]. With increased deformity, chest distortion can limit thoracic cavity space and compliance, predisposing patients to respiratory and cardiac complications [5]. Unfortunately, non-operative management of progressive scoliosis, including bracing, is minimally effective in patients with neuromuscular curves and may cause dermatological, respiratory, and esophageal complications without affecting curve magnitude, shape, or rate of progression [1,5,7]. Consequently, fusion is the definitive treatment for progressive curves in this patient population. Complications from spinal fusion are increased in the context of the multisystem comorbidities of many children with CP, making pre-operative assessment of all individual risk factors and medical optimization crucial [1,8].

To establish standardized pre-operative assessments, identifying all risk factors that predispose patients to complications following surgical treatment is necessary. Known risk factors include pre-existing intracerebral lesions, use of anti-seizure medications, coagulopathies, poor upper airway tone, recurrent aspiration episodes, gastroesophageal reflux disease, feeding problems, non-ambulatory status, and larger curves [1,6]. However, there is a dearth of literature analyzing patient race as a risk factor for post-operative complications. The purpose of this study was to evaluate if race is an independent risk factor for children with CP to experience revision surgery, post-operative complications, and prolonged intensive care unit (ICU) stays following spinal fusion surgery for neuromuscular scoliosis.

## Materials and methods

Data acquisition

A retrospective cohort analysis was conducted using the American College of Surgeons National Surgical Quality Improvement Program (NSQIP) Pediatric database from 2012 to 2019. The NSQIP database contains data on patient demographics, comorbidities, and adverse events following surgical procedures from over 140 sites nationally, a majority being academic institutions. All patient, provider, and hospital information are de-identified [9].

Patient selection

Patients under the age of 18 years who underwent spinal fusion procedures were identified using current procedural terminology (CPT) codes and International Classification of Diseases, Ninth and Tenth Revisions, Clinical Modification (ICD-9-CM, ICD-10-CM) codes. NSQIP lists patient records with a CP indicator, which is defined as “patients who have been diagnosed with CP with associated motor and/or cognitive deficits due to known or unknown etiology” [10]. In addition, if either ICD-9 began with “343” or ICD-10 began with “G80,” patients were included with CP. Patient cases were then filtered to CPT codes related to spinal instrumentation and arthrodesis (22800, 22802, 22804, 22808, 22810, 22812), as well as revision spinal arthrodesis (22852) (Table 1). A total of 3,081 patients met the inclusion criteria for analysis.

**Table 1:** Statistically significant associations between race and characteristics of cerebral palsy patients undergoing procedures for spinal fusion. *P-value is significant. Other races included Hispanic, Asian, Hawaiian and Pacific Islanders, Native American and Alaskan Natives. Comorbidities are included if there are more than five cases. ASA: American Society of Anesthesiologists; IVH: intraventricular hemorrhages

Characteristics		Caucasian, n (%)	Black, n (%)	Other, n (%)	p-Value
n		1,715	636	730	-
Age (years)	<10	192 (11.2)	97 (15.3)	92 (12.6)	0.059
	10-13	570 (33.3)	224 (35.2)	224 (30.8)	
	14-16	650 (38)	219 (34.4)	281 (38.6)	
	>17	299 (17.5)	96 (15.1)	131 (18)	
Gender	Male	862 (50.4)	332 (52.2)	370 (50.8)	0.705
	Female	853 (49.9)	304 (47.8)	360 (49.5)	
BMI	Underweight	656 (38.3)	261 (41)	312 (42.9)	0.056
	Normal weight	850 (49.7)	285 (44.8)	311 (42.7)	
	Overweight	117 (6.8)	50 (7.9)	60 (8.2)	
	Obese	86 (5)	38 (6)	47 (6.5)	
	Under 2	6 (0.4)	2 (0.3)	0 (0)	
Comorbidity	Ventilator	146 (8.5)	56 (8.8)	80 (11)	0.150
	Asthma	352 (20.6)	163 (25.6)	156 (21.4)	0.027*
	Chronic lung disease	275 (16.1)	120 (18.9)	163 (22.4)	<0.001*
	Oxygen supplied	139 (8.1)	43 (6.8)	67 (9.2)	0.262
	Tracheostomy	125 (7.3)	63 (9.9)	57 (7.8)	0.113
	Structural pulmonary abnormalities	282 (16.5)	106 (16.7)	136 (18.7)	0.406
	Esophageal, gastric, or intestinal disease	790 (46.2)	282 (44.3)	316 (43.4)	0.415
	Previous cardiac surgery	115 (6.7)	36 (5.7)	41 (5.6)	0.475
	Impaired cognition status	1,532 (89.5)	588 (92.5)	662 (90.9)	0.070
	Seizure	1,126 (65.8)	443 (69.7)	467 (64.1)	0.074
	IVH	124 (7.2)	49 (7.7)	29 (4)	0.005*
	Nutritional support	852 (49.8)	323 (50.8)	388 (53.3)	0.291
	Cardiovascular event	166 (9.7)	52 (8.2)	49 (6.7)	0.051
	Steroid use within 30 days	31 (1.8)	17 (2.7)	15 (2.1)	0.420
	Weight loss	31 (1.8)	12 (1.9)	4 (0.5)	0.047*
	Low albumin	28 (1.6)	12 (1.9)	12 (1.6)	0.909
	Bleeding disorder	15 (0.9)	5 (0.8)	2 (0.3)	0.264
	Hematological disorder	98 (5.7)	32 (5)	30 (4.1)	0.257
	Inotropic support	25 (1.5)	10 (1.6)	9 (1.2)	0.860
	Malignancy	17 (1)	3 (0.5)	6 (0.8)	0.472
	Open wound	32 (1.9)	12 (1.9)	7 (1)	0.240
	Previous sepsis	11 (0.6)	9 (1.4)	4 (0.5)	0.119
	ASA (≥3)	1,316 (76.9)	494 (77.7)	539 (74)	0.194
Cardiac risk factors	No risk factors	1,481 (86.6)	561 (88.2)	633 (87)	0.590
	Minor risk factors	143 (8.4)	42 (6.6)	61 (8.4)	
	Major risk factors	80 (4.7)	32 (5)	33 (4.5)	
	Severe risk factors	11 (0.6)	1 (0.2)	3 (0.4)	

Patient characteristics

Patient characteristics used in this analysis included gender, race, age, body mass index (BMI), and comorbidities. Race was assigned by grouping patients into either Caucasian, Black, or other. “Other” encompassed Asian, Native Hawaiian or Pacific Islander, American Indian or Alaskan Native, Hispanic ethnicity, or otherwise not specified. Of note, we understand that grouping all these races together is not ideal, however, one major limitation of the NSQIP database is the lack of ability to tease out individual specific races other than Caucasian or Black. Patient age was divided into four ranges based on the mean age (14 years) and standard deviation (three years). In accordance with the Centers for Disease Control and Prevention (CDC) recommendations for pediatrics, BMI range was assigned as the following: underweight (BMI <5th percentile), normal weight (between the 5th and 85th percentiles), overweight (between the 85th and 95th percentiles), and obese (>95th percentile). Due to the extensive number of patient comorbidities listed in the NSQIP database, comorbidities were only included for analysis if there were five or more occurrences of the comorbidity in the sample population. These included ventilator requirements, asthma, history of chronic lung disease, oxygen support, tracheostomy, structural pulmonary abnormalities, esophageal, gastric or intestinal disease, previous cardiac surgery, impaired cognition status, seizure, cardiac risk factors (none, minor, major, severe), cerebrovascular event (including stroke or brain injury), intraventricular hemorrhage, chronic steroid use (within 30 days), nutritional support (parenteral or feeding tubes), weight loss, low albumin, bleeding disorder, hematological disorder, open wound, previous sepsis, childhood malignancy, pre-operative inotropic requirement, and American Society of Anesthesiologist (ASA) classification 3 or greater.

Post-operative complications

Surgical site-related complications included superficial surgical site infection (SSI), deep SSI, organ SSI, superficial wound dehiscence, or deep wound dehiscence. Medical-related complications included pneumonia, intubation, pulmonary embolism, progressive renal insufficiency, acute renal failure, urinary tract infection (UTI), coma, stroke/intracranial hemorrhage, seizure, nerve injury, intraventricular hemorrhage, cardiac arrest, bleeding/transfusions, venous thrombus, *Clostridium difficile* infections, sepsis, septic shock, and central line-associated bloodstream infections. Total complications were defined as the summation of surgical site complications, medical complications, 30-day unplanned readmissions, and unplanned reoperations. ICU length of stay and total length of stay were reported in days and analyzed separately from the previously mentioned complications.

Statistical analysis

IBM SPSS version 28.0 (Chicago, IL: IBM Corp.) was used for analysis. Post-operative complications, need for revision procedures, and length of stay were evaluated for associations with patient race. To control for confounding variables, demographics and comorbidities were evaluated for associations to race with univariate and multivariate analyses. Characteristics with univariate p<0.05 were identified as significantly associated with race and were subsequently controlled for in the multivariate logistic regression analysis with the Hosmer-Lemeshow goodness of fit test. An odds ratio and confidence interval were provided in both adjusted and unadjusted analyses, and Caucasian race was established as the reference in multivariate regression models. Kruskal-Wallis H test was used to analyze race and both ICU and total hospital length of stay post-operatively, with post-hoc pairwise comparisons using the Dunn-Bonferroni approach. ICU admission following spinal fusion surgery is typical for this patient population due to their complex medical comorbidities. Therefore, we excluded cases where ICU and total hospital length of stays were listed as blank or zero. Other race was established as the reference in Dunn-Bonferroni post-hoc pairwise comparison. Significance was assessed at a p-value of <0.05 with two tails.

## Results

Univariate analyses

A total of 3,081 patients with CP under the age of 18 years who underwent spinal fusion were identified, including 1,715 (55.7%) Caucasian, 636 (20.6%) Black, and 730 (23.7%) other patients. The mean age was 14 years; proportionally more Black patients were younger than 14 years of age (321/636, 50.5%) compared to Caucasian (762/1.715, 44.4%) and other patients (316/730, 43.3%), but this difference was not statistically significant (p=0.059). Table 1 shows the demographics and comorbidities stratified by patient race. Black patients had proportionally higher rates of asthma (25.6% vs 20.6% vs 21.4%, p=0.027), intraventricular hemorrhage (7.7% vs 7.2% vs 4%, p=0.005), and weight loss or failure to thrive (1.9% vs 1.8% vs 0.5%, p=0.047) compared to Caucasian and other patients. Other patients had proportionally higher rates of chronic lung disease (22.4% vs 16.1% vs 18.9%, p<0.001) compared to Caucasian and Black patients.

Multivariate analyses

Table 2 lists the associations between race and risk for revision procedure and post-operative complications. There were 59 revision procedures out of 3,081 (1.9%) cases; after controlling for the significant comorbidities identified in the univariate analysis, race was not an independent risk factor for revision spinal fusion. However, both Caucasian (p=0.012) and Black (p=0.003) races were independent risk factors for experiencing any post-operative complication, with Black patients having an increased risk compared to Caucasians (adjusted odds ratio {OR} 1.322, 95% CI 1.099-1.590) (Table 3). Both Caucasian (p=0.005) and Black (p<0.001) races are risk factors for experiencing a medical complication, with Black patients having an increased risk compared to Caucasians (OR 1.373, 95% CI 1.130-1.667). The most common medical complication for approximately 80% of patients across all races was bleeding and/or need for transfusions, followed by post-operative pneumonia in approximately 5% of patients across all races (Table 3). The most common surgical complication was superficial wound dehiscence suffered by approximately 4% of patients in each race group; the second most common was superficial incisional infection for Caucasian and other patients (2.3% vs 2.2%) and deep incisional infection for Black patients (3.0%) (Table 3). There were no significant associations between race and risk of surgical site complications, 30-day unplanned readmissions, or revision fusion. There was a statistically significant difference in post-operative ICU (H{2}=6.706, p=0.035) and total hospital (H{2}=38.068, p<0.001) length of stay between race groups (Table 4). The other race group had both a statistically greater length of ICU stay than Caucasians (Mdn=3.00 vs Mdn=2.00 days, p=0.029) and a statistically greater length of total hospital stay than Caucasians and Blacks (Mdn=9.00 vs Mdn=6.00 vs Mdn=6.00 days, p<0.001) (Table 4). No other differences were statistically significant.

**Table 2:** Multivariate analysis of race on revision procedures and post-operative complications for spinal fusion. *P-value is significant.

Variables	95% CI					Hosmer-Lemeshow
	N (%)	Odds ratio	Lower	Upper	p-Value	
Revision procedure	59 (1.9)	-	-	-	-	-
Caucasian	38 (2.2)	REF	REF	REF	0.163	0.910
Black	13 (2)	0.92	0.486	1.742	0.797	-
Other	8 (1.1)	0.475	0.22	1.025	0.058	-
Any complication	1,252 (40.6)	-	-	-	-	-
Caucasian	663 (38.7)	REF	REF	REF	0.012*	0.722
Black	290 (45.6)	1.322	1.099	1.590	0.003*	-
Other	299 (41)	1.085	0.908	1.296	0.37	-
Any surgical site complication	182 (5.9)	-	-	-	-	-
Caucasian	104 (6.1)	REF	REF	REF	0.891	0.994
Black	36 (5.7)	0.919	0.622	1.360	0.673	-
Other	42 (5.8)	0.938	0.648	1.360	0.737	-
Any medical complication	957 (31.1)	-	-	-	-	-
Caucasian	493 (28.7)	REF	REF	REF	0.005*	0.718
Black	227 (35.7)	1.373	1.130	1.667	<0.001*	-
Other	237 (32.5)	1.170	0.969	1.412	0.102	-
Any unplanned readmission	294 (9.5)	-	-	-	-	-
Caucasian	162 (9.4)	REF	REF	REF	0.868	0.889
Black	64 (10.1)	1.069	0.788	1.451	0.667	-
Other	68 (9.3)	0.973	0.722	1.313	0.860	-
Any unplanned reoperation	207 (6.7)	-	-	-	-	-
Caucasian	126 (7.3)	REF	REF	REF	0.202	0.972
Black	43 (6.8)	0.914	0.488	1.033	0.624	-
Other	38 (5.2)	0.710	0.638	1.310	0.074	-

**Table 3:** Top 3 most common medical and surgical site complications following spinal fusion by race.

Medical complication				Surgical site complication		
Race	First (n, %)	Second (n, %)	Third (n, %)	First (n, %)	Second (n, %)	Third (n, %)
Caucasian	Bleeding/transfusion (1,342, 78.3)	Pneumonia (75, 4.4)	Reintubation (62, 3.6)	Superficial wound dehiscence (66, 3.8)	Superficial incisional infection (39, 2.3)	Deep incisional infection (36, 2.1)
Black	Bleeding/transfusion (514, 80.8)	Pneumonia (30, 4.7)	Sepsis (23, 3.6)	Superficial wound dehiscence (23, 3.6)	Deep incisional infection (19, 3.0)	Organ infection (9, 1.4)
Other	Bleeding/transfusion (586, 80.3)	Pneumonia (37, 5.1)	Urinary infection (26, 3.6)	Superficial wound dehiscence (27, 3.7)	Superficial incisional infection (16, 2.2)	Deep incisional infection (10, 1.4)

**Table 4:** Comparison of length of stay (days) following spinal fusion by race. *P-value is significant. **Significant difference in median per Kruskal Wallis (H{2} 6.706, p=0.035). ***Significant difference in median per Kruskal Wallis (H{2} 38.068, p<0.001). The cases where length of stay was listed as null or zero were excluded.

Race	n	Mean	SD	Pairwise comparison	Median
ICU stay	1,433	3.63	4.028	-	2.00
Caucasian	740	3.45	3.905	p=0.029*	2.00
Black	317	3.71	4.394	p=0.133	2.00
Other	376	3.92	3.938	REF	3.00**
Total stay	3,020	8.06	7.725	-	6.00
Caucasian	1,685	7.73	7.726	p≤0.001*	6.00
Black	627	7.90	7.708	p≤0.001*	6.00
Other	708	9.00	7.674	REF	7.00***

## Discussion

Neuromuscular scoliosis is a common and potentially life-threatening sequela of CP that often requires surgical intervention. Spine fusion is indicated to improve deformity and stop curve progression, level the pelvis, and improve comfort and sitting balance [11]. However, the decision to undergo spinal fusion must be weighed against serious adverse risks including major blood loss, deep wound infection, and unplanned reoperation [11]. Knowledge of comorbidities and characteristics that predispose patients to complications improves our understanding of which patients are appropriate candidates for surgery and informs shared decision-making and management with the patient, family, and other medical teams [12]. The present study identified Black race as an independent risk factor for increased medical and total complications following spinal fusion in patients with CP.

Our findings are consistent with growing evidence across medical and social sciences exploring racial differences in post-operative outcomes between Black and Caucasian patients. Studies have shown that Black and Hispanic children have different rates of surgical intervention than Caucasian patients and higher risk of post-operative complications and mortality when surgery is performed [13,14]. Perhaps most significantly, our findings mirror that of a prior evaluation of the pediatric NSQIP database that found Black patients have higher post-operative morbidity and mortality compared to Caucasians across all surgery types [15]. Consideration of race as an independent risk factor for post-operative complications in children with CP is helpful in pre-operative risk assessments, and for families and other medical providers assisting in post-fusion patient care to appreciate each patient’s individualized risks. The underlying causes of these discrepancies require further study, though our findings are likely at least partly attributed to the higher comorbidity burden in Black patients [15,16]. Differences in physician-patient relationships and communication, bias, access to health resources, and patient income have also been proposed as underlying factors for varying outcomes based on race [14,16]. Recognizing race as a risk factor in pediatric patients with CP and neuromuscular scoliosis is the first step in developing concrete recommendations on mitigating the impact of this risk when spine fusion is pursued.

This study also found that “other” races had statistically significant greater lengths of stay in the ICU than Caucasian patients, and statistically significant greater total hospital stay compared to both Caucasian and Black patients. This could be due to delays in translator services in the peri-operative setting or other independent risk factors. Studies have shown that pre-operative risk factors and surgical risk factors predict hospital costs more effectively than post-operative complications [17]. Pre-operative assessment may help to mitigate factors that increase length of stay in the ICU, which can ultimately reduce the costs for both the hospital and the patients.

There are several limitations to this study. NSQIP is built on non-randomized sampling; participating hospitals decide which cases to submit to the database while maintaining quotas for certain surgery types. It is possible that miscoding or data entry errors could have led to inaccurate reporting of results. NSQIP does not include GMFCS class of the patients, which could be an underlying clinical confounding variable. NSQIP does not provide information on the treating institution, which may be an important confounder if some institutions were more likely to treat different race groups, and have different resources or protocols regarding important aspects of patient care. Additionally, only the first 30 days following surgery are tracked for post-operative outcomes except for total hospital stay. Re-admission and ICU stay beyond this time frame were unable to be determined. Finally, there could be other social confounding variables contributing to these differences that cannot be accounted for by the NSQIP database.

## Conclusions

This is the first study to evaluate race as a risk factor for post-operative complications following spinal fusion for neuromuscular scoliosis in children with cerebral palsy. Based on this database review, Black patients were at an increased risk of post-operative medical complications. Non-Caucasian and non-Black patients had significantly greater ICU and total hospital stays post-operatively. Whether these differences are due to unrecognized confounding variables or systemic bias will be the topic of further study. Further investigation and enhanced awareness will be helpful in improving post-operative patient care and reducing hospital costs associated with spine fusion in children with CP.
